# Foreign Summary

**Published:** 1838-11-07

**Authors:** 


### FOREIGN SUMMARY.

*Modification of the Appareil Immobile, for the Treatment of Fractures.*—In No. 21, we gave a description by M. Velpeau, of the *appareil immobile*, of M. Seutin, for the treatment of fractures. From the London Medical Gazette for August 11th, we take the following suggestions for the improvement of this apparatus, by Drs. Christophers and King. The objection to M. Seutin's apparatus is, that it does not expand and contract as the limb may swell or diminish with the development or subsidence of inflammation. Dr. King's first idea was to slit the apparatus, so that it might yield and return upon itself according to the variation in the volume of the limb. It answered the purpose tolerably well, but was not sufficiently elastic to follow the limb in its changes of volume. At the suggestion of Dr. Christophers, a further improvement was now adopted, which consists in a simple and ingenious contrivance. "He proposed applying around the apparatus slit open, a certain number of elastic straps, made of India rubber, with buckles which admit of their being drawn to the requisite tightness. They are rather more than an inch wide, and rather longer than is necessary to encompass the limb. Four of these were applied, and converted the apparatus into a case sufficiently elastic to follow the changes in the volume of the limb, and yet of sufficient strength to afford the requisite support. Seutin's apparatus, thus modified, fulfils, as nearly as possible, and much better than any other, all the indications required; and it must be evident that it will be even a greater boon to the patient affected with a compound fracture than to one whose fracture is simple.

In case the limb undergoes a considerable diminution of volume, it will only be necessary to remove a longitudinal strip of the apparatus, instead of opening it by a longitudinal incision; and the strip should, of course, be removed, or the slit made along that side of the limb on which the nerves and vessels exist, and which can least bear pressure. We deem it not improbable that the apparatus, thus modified, will be found useful in the treatment of many diseases, where it is essential to keep the parts motionless, without exercising an unyielding resistance, or a pressure in the least degree unequal. Dr. Christophers proposes to employ it for that troublesome disease—a varicose state of the veins."

*Treatment of Varicose Veins.*—A writer in the London Medical Gazette, recommends the introduction of needles *with a cutting edge*, and a small, round silk ligature under the vessels. Commenting on the report of the Pennsylvania hospital operations for the cure of varicose veins, copied into the British Journals from the Examiner, he remarks that "the passing of a needle *through*, as well as under the vessels, before applying the ligature, does not seem to have any advantage over the latter mode, if the needle has a cutting edge, and the thread is round, small, and firmly applied."

*Nitrate of Strychnine for Paralysis.*—A child, three and a half years of age, born of healthy parents, remained apparently well until the end of April, 1834, when, without any apparent cause, it was seized with paralysis and convulsive movements of the upper and lower extremities, and paralysis of the tongue; the expression of the face was wild, and the child had been in this state fourteen days when the author was called in; he found no symptoms of fever or congestion about the head; the appetite was good; tongue clean; bowels natural. As the child had formerly passed some worms, anthelmintics were administered, and a few lumbrici expelled, but without any relief; on the contrary, the child became thinner every day. The following medicine was now given:—

*Nitrate of Strychnine*, gr. i.; dissolve in
*Alcohol*, one drachm; add
*Cinnamon water*, two drachms. Three drops thrice a day.

The dose was gradually increased until the child took 30 drops or 1-10th grain of strychnine in the course of the day.

After the lapse of a few days the patient's condition was much improved, the convulsive movements declined, and the power over the extremities was gradually recovered, and at the end of six weeks the child was completely cured.—*Lancet, from Sieb. Journal*, Vol. 17, No. 3.

Fifty-four surgeons of the Royal Navy have accepted the commuted allowance offered by the Admiralty, and will be placed on the retired list.

